# The Progress on Genetic Analysis of Nasopharyngeal Carcinoma

**DOI:** 10.1155/2007/57513

**Published:** 2008-01-21

**Authors:** Xiaofeng Zhou, Jing Cui, Virgilia Macias, André A. Kajdacsy-Balla, Hui Ye, Jianguang Wang, P. Nagesh Rao

**Affiliations:** ^1^Center for Molecular Biology of Oral Diseases, College of Dentistry, Graduate College, UIC Cancer Center, University of Illinois at Chicago, Chicago, IL 60612, USA; ^2^Guanghua School & Research Institute of Stomatology, Sun Yat-Sen University, Guangzhou 510055, China; ^3^Department of Medicine, Brigham and Women's Hospital, Harvard Medical School, Boston, MA 02115, USA; ^4^Department of Pathology, College of Medicine, University of Illinois at Chicago, Chicago, IL 60612, USA; ^5^Xinhua Hospital, Shanghai Children's Medical Center, Shanghai Jiaotong University, Shanghai 200127, China; ^6^Department of Oral & Maxillofacial Surgery, The Second Affiliated Hospital of Sun Yat-Sen University, Guangzhou 510120, China; ^7^Department of Pathology & Laboratory Medicine, David Geffen School of Medicine, University of California at Los Angeles, Los Angeles, CA 90095, USA

## Abstract

Nasopharyngeal carcinoma (NPC) is a rare malignancy in most parts of the world, but is one of the most common cancers in Southeast Asia. Both genetic and environmental factors contribute to the tumorigenesis of NPC, most notably the consumption of certain salted food items and Epstein-Barr virus infection. This review will focus on the current progress of the genetic analysis of NPC (genetic susceptibilities and somatic alterations). We will review the current advances in genomic technologies and their shaping of the future direction of NPC research.

## 1. INTRODUCTION TO NPC

The nasopharyngeal carcinoma (NPC) is a malignancy of the head and neck region that arises from the epithelial cells that cover the surface and line the nasopharynx. This disease was initially reported in 1901, and characterized clinically in 1922 [1]. It is a rare malignancy in the United States, accounting for 2% of all head and neck squamous cell carcinomas, with an incidence of 0.5 to 2 per 100,000. However, it is endemic in many geographical regions, including Southern China and Southeast Asia, where the observed incidence rates range from 15 and 50 per 100,000 persons. An intermediate incidence has been reported in Alaskan Eskimos and in the Mediterranean basin (North Africa, Southern Italy, Greece, and Turkey), ranging from 15 to 20 cases per 100,000 persons [2]. A male preponderance exists; with a male-to-female ratio of approximately 2:1. Overall, NPC can occur in all age groups, but has a bimodal age distribution. The incidence peaks at 50 to 60 years of age; and a small peak is observed during late childhood [3].

### 1.1. Anatomy

**Figure 1:**
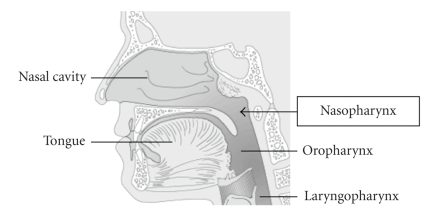
Anatomic site of NPC.

The nasopharynx (the upper part of the throat, behind the nose) is a cuboidal chamber (about 1.5 inches on each edge) located posterior to the nasal choanae (see Figure 1). It is bounded superiorly by the clivus, and inferiorly by the lower border of the soft palate. The posterior border is made up by the mucosa that overlies the superior constrictor muscles of the pharynx and the prevertebral fascia of the C1 and C2 vertebral bodies. Its lateral walls contain the Eustachian tubes' orifices. The fossa of Rosenmüller represents the most common site of origin for NPC [4].

### 1.2. Epidemiology

In endemic regions, NPC presents as a complex disease caused by an interaction of the oncogenic gamma-herpesvirus Epstein-Barr virus (EBV) chronic infection, environmental, and genetic factors, in a multistep carcinogenic process. The EBV is spread worldwide, infecting over 95% of the adult population [5]. It is transmitted by saliva and its primary infection occurs during childhood with replication of the virus in the oropharyngeal lining cells, followed by a latent infection of B lymphocytes (primary target of the EBV). Although the infection is typically subclinical, the virus is associated with later development of several malignancies, including NPC [6]. Elevated titers of EBV associated antigens (especially of IgA class), a latent EBV infection identified in neoplastic cells of virtually all cases of NPC, and the clonal EBV genome consistently detected in invasive carcinomas and high-grade dysplastic lesions suggest a critical role of EBV in the pathogenesis of NPC in endemic areas.

Significant environmental factors contribute to NPC include the consumption of foods high in salt, exposure to nitrosamines and polycyclic hydrocarbons as important carcinogens. In nonendemic areas, the association of NPC with alcohol and tobacco use has been reported, either as weak or controversial in some series [3, 7].

Genetic studies of endemic populations revealed the association of HLA antigen haplotype with NPC: HLA-2, HLA-B17, and HLA-Bw26 double the risk of the disease, and genomic and cytogenetic studies have shown multiple aberrations in chromosomes 1, 3, 9, 11, 12, and 14. These genetic factors will be discussed in association with the current advances in genomic technologies in the following sections.

### 1.3. Sign and symptoms

NPC may easily escape diagnosis at early stages, and most of the cases remain undiagnosed until they present as a metastasis to the lymph nodes of the neck. The tumor is difficult to diagnose for multiple reasons including the nonspecificity of the initial symptoms and the difficulty of examining the postnasal space. Additionally, lesions can grow within the submucosa of the nasopharynx and escape endoscopic visualization [8–10]. The majority of tumors arise in the lateral walls, especially from the fossa of Rosenmuller and Eustachian tube cushions. Tumors can grow within the nasopharynx or extend to the opposite lateral wall; they can also infiltrate other structures toward the base of the skull, and invade the palate, nasal cavity, or the oropharynx. The most common presenting symptom is a painless cervical lymph node enlargement due metastasis, followed by nasal, aural, and neurological symptoms. A unilateral neck mass is reported in about 36% of cases, but other series report rates as high as 80% [3]. Only 5% of cases reported in Southern China present with distant metastases [2]. Enlargement and extension of the tumor within the nasopharynx may cause nasal obstruction-related symptoms such as congestion, nasal discharge, and bleeding. Blockage of the Eustachian tube and/or extension into the ear may result in changes in hearing or hearing loss (usually unilateral). Extension of the tumor into the base of the skull is usually associated with cranial nerve deficits. The most common distal metastatic sites are bone, lung, mediastinum, and more rarely liver [11]. Symptoms related with the distal metastatic disease include bone pain or organ dysfunction.

### 1.4. Pathology

With the constant advance in our understanding of this disease, the pathohistological classification of NPC has been evolving continuously. In 1978, the histological classification guideline proposed by the World Health Organization (WHO) categorized NPC into three groups: type 1 (keratinizing squamous cell carcinoma), type 2 (nonkeratinizing carcinoma), and type 3 (undifferentiated carcinoma). Types 2 and 3 have also been called lymphoepithelioma [1, 3]. The 1991 WHO classification of nasopharyngeal carcinomas divided them into two groups: squamous cell carcinoma (keratinizing squamous cell carcinoma, type 1 of the former classification), and nonkeratinizing carcinoma (types 2 and 3 of the former classification combined under a single category). The second group (nonkeratinizing carcinoma) was further subdivided into differentiated and undifferentiated carcinomas. Lymphoepithelioma-like carcinoma was considered a morphologic variant of undifferentiated carcinoma [1]. The current WHO classification keeps the 1991 terminology, and adds one additional category: basaloid squamous cell carcinoma [1, 12].

Published data indicate a probably higher proportion of keratinizing squamous cell carcinoma among all NPC in nonendemic areas compared with endemic areas. Some studies reported that squamous cell carcinoma (former WHO type 1) accounts for approximately 25% of all NPC in North America, but only 1% in endemic areas; whereas undifferentiated carcinoma (former WHO type 3) accounts for 95% of all cases in high incidence areas, but 60% of cases in North America [1, 3, 12].

### 1.5. Staging

The extent of the disease is the most important prognostic factor, and staging will have a great impact on the selection of treatment in patients with NPC [1]. The tumor-node-metastasis (TNM) staging system, promulgated by the American Joint Comittee on Cancer (AJCC), is the most frequent system used to classify the extent of spread of nasopharyngeal carcinomas [13]. Information about the tumor, lymph nodes, and metastasis is combined according to a process called stage grouping. Each set use Roman numerals O to IV to describe progression from earliest to most advanced stage. Therefore, according to this system, patients are designated into stages 0, I, IIA, IIB, III, IVA, IVB, and IVC [12, 13].

### 1.6. Diagnosis

NPC shows an extraordinarily high cure rate for early stage disease, thus early detection is critical to improve the overall prognosis and reduce morbidity and metastasis [10]. The detection of NPC is based on the clinical history and the physical examination, but a definitive diagnosis requires a biopsy of the lesion [3]. A series of radiologic tests, including a computed tomography (CT) scans with intravenous contrast and magnetic resonance imaging (MRI) of the head and neck are currently being used to assess the tumor extension and the stage of the disease [11].

EBV-related antigens in sera are also useful markers for NPC diagnosis [7, 14]. Ho et al. found an increased diagnostic sensitivity and specificity (99% and 96%, resp.) using a combination of serum protein profiles with an EBV antibody serology test [15]. A clinical history of a known metastasis but an unknown site of primary tumor with a positive serology for EBV may also help in diagnosis, redirecting the search for a primary disease at the nasopharynx. The plasma Epstein-Barr virus DNA (EBV-DNA) level has also been suggested to be a reliable indicator for staging and prognosis of NPC [16]. The EBV infection can also be detected by immunostaining of tumoral cells for latent membrane protein 1 (LMP-1), and/or in situ hybridization for EBV-encoded RNAs. Results using these techniques on paraffin-embedded tissue sections support the evidence that EBV plays a major role in the pathogenesis of the disease [17].

### 1.7. Treatment

External radiotherapy alone is still the primary treatment for early stage NPC. Concomitant chemoradiotherapy has been used in recent years for locally advanced disease. The management of recurrent cervical lymph node metastases in NPC after radiation and chemotherapy is a radical surgery of the lymph nodes of the neck with postoperative brachytherapy. The overall 5-year survival rate for patients with locally advanced disease is around 55–60%. The salvage surgical procedure for persistent or recurrent neck disease shows a 5-year control rate of 66% and a 5-year actuarial survival of 38% [18, 19].

## 2. GENETIC ANALYSES OF NPC

While nasopharyngeal carcinoma is a rare malignancy in most parts of the world, it is one of the most common cancers in Southeast Asia including areas such as southern China, Hong Kong, Singapore, Malaysia, and Taiwan. The reported incidence in these countries ranges from 10 to 53 cases per 100,000 persons. The incidence is also high among Eskimos in Alaska and Greenland and in Tunisians, ranging from 15 to 20 cases per persons [2]. A clear and specific etiology for NPC is still lacking. In general, NPC is thought to be the result of both genetic susceptibility and environmental factors, such as consumption of certain salted food items [20] and infection with EBV [21]. Familial clustering of NPC has been widely observed in both the Chinese population [22, 23], and non-Chinese patient cohort [24]. The familial risk of NPC is among the highest of any malignancy [25]. The described relative risk of NPC in first-degree relatives is about 8.0 [26, 27]. In this article, we will review the current progress on genetic analysis of nasopharyngeal carcinoma (e.g., genetic susceptibilities and somatic alterations) in relationship with recent advances in genomic technologies.

### 2.1. Progress on searching for genetic susceptibilities of NPC

Although perhaps not Mendelian, strong evidences suggest that genetic factors play important roles in NPC. Epidemiological studies suggest that most of the familial aggregation of NPC derives from inherited susceptibility [2]. A recent complex segregation analysis on a Chinese cohort provided additional evidence to support a multifactorial mode of inheritance for NPC [28]. However, the molecular genetic basis of NPC remains unknown. Most of the studies searching for the susceptibility genes of NPC can be loosely categorized into 2 methodologies: a positional cloning approach and a functional cloning approach. A positional cloning approach aims first to identify the genomic location (or locus) that is linked to the disease. This is followed by the identification of the disease gene (or susceptibility gene) at this particular genomic location. The functional cloning approach, also known as candidate gene-based approach, requires sufficient prior knowledge of the disease and the functional defect(s) associated with the disease. Candidate gene(s) are identified based on this knowledge. Mutations (or polymorphisms) will then be identified and investigated in the candidate gene. These approaches complement each other. Positional cloning approaches can lead to identification of a candidate gene for functional cloning studies. On the other hand, a functional cloning approach often confirms the genomic location of the susceptibility locus identified by positional cloning studies. The following sections will summarize the progress of identifying the NPC susceptibility genes based on these approaches.

#### 2.1.1. Positional cloning approach searching for NPC susceptibility genes

Linkage analyses are the most common approaches for the identification of a disease locus (or susceptibility locus). There are several variations of linkage analysis design, based on the pedigree structure. The linkage studies usually involve genotyping of both affected individuals and healthy family members using a panel of genetic markers. Most of the linkage studies on NPC performed so far have used microsatellite markers that are essentially polymorphic tandem repeats of di- to tetranucleotide sequence motifs flanked by unique sequences. This approach is usually tedious, labor-intensive, and requires large amounts of sample DNA, allowing only a modest number of markers to be screened. However, the recent completion of the human genome project has lead to the identification of millions of single nucleotide polymorphisms (SNP), the most abundant type of polymorphism in the human genome, which will lead to another wave of intense search for the NPC susceptibility locus/gene.

Several linkage analyses studies suggested the association of susceptibility HLA haplotypes with NPC development. Most studies conducted among the Chinese population demonstrated an increased risk of NPC for individuals with HLA-A2. A recent study detected a consistent association between NPC and the prevalent Chinese HLA-A2 subtype (HLA-A*0207), but not the prevalent Caucasian subtype (HLA-A*0201) [29]. The HLA types of AW19, BW46, and B17 have also been reported to be associated with an increased risk, whereas HLA-A11 is associated with a decreased risk [30]. The involvement of HLA in NPC tumorigenesis may be through its cytotoxic T cell recognition and host immune response to EBV infection. However, it has been suspected that HLA alleles may not directly contribute to the susceptibility of NPC. Interestingly, Lu et al. (1990) reported a linkage study based on affected sib pairs which suggested that a gene closely linked to the major histocompatibility complex (MHC) region but distinct from the HLA genes confers a greatly increased risk of nasopharyngeal carcinoma [31].

A recent study provides evidence for the linkage of NPC to chromosome 3p and a fine map of NPC susceptibility locus to a 13.6-cM region on 3p21.31-21.2 [32]. These results are in agreement with several previous studies that suggest that the deletion of chromosomes 3p is a common genetic event in NPC [33, 34]. Many tumor suppressor candidate genes such as CACNA2D2, DLC1, FUS1, H37, HYAL1, RASSF1A, SEMA3B, and SEMA3F and tumor susceptibility genes such as hMLH1 have been isolated from the region [32]. These studies indicate that genes in the 3p21 may play a critical role in tumorigenesis of familial NPC. Consistent with this notion, another study detected a high frequency of loss of heterozygosity on 3p, in histologically normal nasopharyngeal epithelia and dysplastic lesions from Southern Chinese individuals, suggesting that the genetic abnormality appear to be causative for NPC [35]. Isolation and identification of susceptibility genes from 3p21 may greatly advance the understanding of the etiology and development of NPC.

A recent genome-wide scanning of 20 families with included 65 affected individuals provides evidence of a major susceptibility locus for NPC on chromosome 4p15.1-q12 [36]. The strongest linkage was observed with marker D4S405 (LOD score = 3.54) and D4S3002 (LOD score = 4.2). Interestingly, when EBV antibody titer was included as a covariate, the LOD scores reached 4.70 and 5.36 for these markers, respectively. This observation was recently confirmed by a population-based large-scale study of Han Chinese from Guangxi province using 34 microsatellites spanning an 18-megabase region of chromosome 4 (4p15.1-q12) [37].

#### 2.1.2. Functional cloning approach searching for NPC susceptibility genes

Recent studies suggested that genetic polymorphisms in genes that metabolize carcinogens are associated with NPC susceptibility. Cytochrome P450 2E1 (CYP2E1) is one of the cytochrome P450s and is responsible for the metabolic activation of nitrosamines and the related carcinogens. The variant form of CYP2E1 has a marked difference in its activity and causes different levels of DNA damage in human cells. Nitrosamines are the effective carcinogens for NPC and are believed to be involved in the pathogenesis of NPC. Case-control studies have shown a strong association of the variant form of CYP2E1 (c2 allele) with increased risk of this disease in Chinese populations [38, 39]. Other nitrosamine metabolizing genes, such as Cytochrome P450 2A6 (CYP2A6), have also been suggested to play a role in NPC susceptibility [40].

Genetic polymorphism of glutathione S-transferase M1 (GSTM1) is a phase II enzyme known to play an important role in the detoxification of several carcinogens found in tobacco smoke, a synergistic risk factor for NPC [41]. This enzyme also modulates the induction of other enzymes and proteins that are important for cellular functions, such as DNA repair. The enzyme is therefore important to metabolize carcinogens, maintaining genomic integrity and cancer susceptibility. A recent study in the United States has reported that GSTM1 null genotype is associated with an almost twofold increase in risk for NPC [42]. The findings implied that polymorphisms of this modifier might lead to different cellular responses to environmental carcinogens among different individuals, different degrees of genetic instability or damages in the nasopharyngeal epithelial cells. Similar associations were observed in studies on Tunisian and Thai populations [43, 44].

The association of other DNA repair genes with NPC susceptibility has also been implied. Both X-ray repair cross-complementing group 1 gene (XRCC1) and 8-oxoguanine glycosylase 1 (hOGG1) are important in DNA base excision repair. While a reduced risk for NPC was observed with polymorphism of the XRCC1 gene (Arg280His), polymorphism of the hOGG1 gene (Ser326Cys) was shown to be associated with an increased risk for NPC in the Taiwan population [45]. The reduced risk of NPC associated with polymorphism in the XRCC1 gene was confirmed with a different polymorphism (Arg194Trp) recently identified in the population from Guangdong, China, particularly in males and smokers [46]. Interestingly, the higher risk of NPC was observed among those subjects with certain combined genotypes for both hOGG1 and XRCC1 polymorphisms [45], clearly suggesting that carriers of multiple putative high-risk genotypes have the highest risk of developing NPC.

The potential roles of genes that contribute to the immune response have also been studied. Signaling pathways activated by the toll-like receptor 4 (TLR4) involve the induction of anticancer immunity. Functional analyses of an SNP variant of the TLR4 gene at the $3^{\prime }$-untranslated region ($3^{\prime }$-UTR) suggested that it is associated with decreased mRNA stability, and leads to a reduced expression of this gene [47]. This $3^{\prime }$-UTR polymorphism has been shown to be associated with a significantly increased risk for NPC. It is hypothesized that this polymorphism downregulates TLR4 expression through destabilizing the mRNA, and leads in EBV metainfective antiviral immunologic deficits and a high risk of NPC. Similarly, associations with increased risks for NPC have also been detected with polymorphism in toll-like receptor 1, 6, 10, respectively [23, 48].

The palate, lung, and nasal epithelium carcinoma-associated (PLUNC) protein gene plays a role in the innate immune response in the regions of the oral and nasal cavities. In a recent case-controlled study of Chinese population composed of 239 unrelated NPC patients and 286 healthy controls, SNPs in the promoter region of this gene (PLUNC) were significantly associated with susceptibility to NPC, [49]. These results suggest that genetic variation in PLUNC may influence susceptibility to NPC in this Chinese population.

Tremendous enthusiasm in the genetics community has been generated for the identification of millions of polymorphisms (e.g., SNPs) throughout the human genome. Recently, an increasing number of studies have been devoted to investigate the polymorphisms in a variety of cancer-related genes for their potential influence on NPC susceptibility, including matrix metalloproteinases (MMPs) [50, 51], transforming growth factor-beta1 (TGF-beta1) [52], interleukin-10 (IL-10) [53], antigen processing 1 gene (TAP1) [54], p53 [55], cyclin D1 (CCND1) [56], FAS (CD95) [57], mouse double minute 2 (MDM2) [58], and Nedd4 binding protein 2 (N4BP2) [59]. While polymorphisms in these genes have been associated with a statistically significantly increased risk of NPC, the risks are generally small and appear to be restricted to specific studies. It is apparent that the understanding of interactions of these polymorphisms and other risk factors are more important. With the continuous advances in high-throughput sequence and genotyping technologies, this list will increase rapidly.

SNPs appear to be the most abundant sequence variations between individuals. Enthusiasm for very high density SNP sets in the human genome has been largely centered on the potential use for association studies, especially in the context of measured linkage disequilibrium. Indeed, successful implementations using genome-wide association analysis have already been reported for cancer risks [60, 61]. A recent milestone publication by the Welcome Trust Case Control Consortium [62] established the “standard” for genome-wide association analysis, in term of result interpretation, quality control, population stratification, and control sample sharing. These advances in analytical approaches, together with the advent of rapid, affordable, large-scale genotyping methods that enable the cotyping of over 500,000 SNPs on each genomic sample (http://www.affymetrix.com), greatly facilitate the search for new susceptibility genes of NPC, and will lead to a better understanding of the potential interactions among susceptibility genes and between susceptibility genes and environmental factors.

### 2.2. Progress on profiling somatic abnormalities of the NPC genome

Tumors develop through the combined processes of genetic instability and selection, resulting in clonal expansion of cells that have accumulated the most advantageous set of genetic aberrations. Many types of instability can contribute to neoplastic development, including point mutations, chromosomal rearrangements, DNA dosage abnormalities (amplifications or deletions), alteration of microsatellite sequences, and epigenetic changes. Knowledge of genomic aberrations can have clinical implications in diagnosis, treatment, and prognostics of cancer. Four decades ago, the milestone discovery of Philadelphia chromosome (a translocation between chromosome 9 and 22, which fuses the Bcr gene and the Abl tyrosine kinase gene) [63] led to one of the first effective targeted therapies for cancer: treatment of chronic myelogenous leukemia (CML) with the tyrosine kinase inhibitor imatinib (Gleevec). Since then, many exciting clinical advances have been made based on the increasing knowledge of the tumor genome.

During the 1970s and 1980s, several genome-wide approaches were developed to measure these tumor genomic alterations including loss of heterozygosity analysis (LOH) and comparative genomic hybridization (CGH). Advances in genetics and bioengineering have refined these techniques over the past two decades, and the recent development of multicolor staining-based cytogenetic techniques such as multicolor fluorescence in situ hybridization (M-FISH) and spectral karyotyping (SKY) have further improved the ability to analyze the tumor genome [64]. The completion of the human genome project [65, 66] now makes it possible to query the cancer genome systematically in ways that were hitherto impossible. Microarrays designed to analyze targeted genomic regions relevant to chronic lymphocytic leukemia have been produced for use in clinical trials to determine the relationship between therapeutic options and genomic aberrations [67]. Association of genomic aberrations with prognosis has been found for a variety of tumor types, including prostate cancer [68], breast cancer [69], gastric cancer [70], head and neck cancer [71], lymphoma [72, 73], and NPC [74].

#### 2.2.1. Progress on genomic profiling of NPC

**Copy number analysis of NPC**

Comparative genomic hybridization (CGH) was developed to survey gene copy number abnormalities (amplifications and deletions) across a whole genome [75]. In a typical CGH analysis, fluorescently labeled disease DNA (frequently Fluorescein or FITC) and normal DNA (frequently Rhodamine or Texas Red) are cohybridized to the normal metaphase chromosomes to generate fluorescence ratios along the length of chromosomes that provide a cytogenetic representation of DNA copy number variation. CGH was the first effective approach to scanning the entire genome for variations in DNA content [76, 77]. A large number of CGH-based studies on NPC lead to the identification of consistent gain at chromosome 1q, 3q, 8q, 12 and loss at 3p, 9p, 11q, 14q [78–81]. A recent large-scale meta-analysis of CGH results revealed several genomic “hotspots” that show consistent copy number alterations in NPC [82]. These findings provided foundation for further identifications of the corresponding oncogenes and tumor suppressor genes in NPC.

While chromosome-based CGH provided critical hints for identifying candidate genes for NPC, it has a limited mapping resolution (∼20 Mb). Array-based CGH is a second-generation approach in which fluorescence ratios on microarrayed DNA elements provide a locus-by-locus measure of gene copy number variation [83, 84]. Using this approach, frequent amplifications were detected for several oncogene loci, including MYCL1 at 1p34.3 (66.7%), TERC at 3q26.3 (46.7%), ESR at 6q25.1 (46.7%), and PIK3CA at 3q26.3 (40%) [85].

Although the array-based CGH can potentially increase mapping resolution, most of the early arrays used for the CGH studies have utilized large genomic clones, for example, bacterial artificial chromosomes (BACs), which have a limited spatial sensitivity. In addition, large genomic clones also suffer from reduced specificity due to their inclusion of common repeats (e.g., *Alu* and long interspersed nuclear elements (LINEs)), redundant sequences (e.g., low copy repeats (LCRs), also known as segmental duplications), and segments of extensive sequence similarity (pseudogenes or paralogous genes) [86]. Recently, several additional higher-density tools for CGH analysis have become available with the completion of the human genome sequence. These include cDNA array-based CGH [87, 88], oligonucleotide array-based CGH [89, 90], tiling array-based CGH [84], and copy number analysis using high-density SNP microarrays [91–94]. Tiling and SNP array-based approaches have drawn most attention due to their high resolution. Tiling arrays have the potential to resolve small (gene level) gains and losses (resolution ∼40 kb) that might be missed by marker-based genomic arrays which contain large number of gaps due to the distance between the targeted probes [84, 95]. We can envision that in the near future, we will have the ability to survey copy number changes at close to bp resolution using tiling arrays that contain billions of overlapping probes covering the entire genome. The SNP array-based approach provides the unique advantage of concurrent CGH and LOH analysis, which we discuss in further detail below [92, 93].

**Loss of heterozygosity (LOH) analysis of NPC**

Chromosomal aberrations include segments of allelic imbalance identifiable by loss of heterozygosity (LOH) at polymorphic loci, which can be used to identify regions harboring tumor suppressor genes. Allelic losses, which are caused by mitotic recombination, gene conversion, or nondisjunction cannot be detected by CGH and thus require LOH analysis for their identification. This approach is “favored” by the Knudson two-hit hypothesis [96, 97] for hunting the tumor-suppressor genes. Traditionally, polymorphic markers, such as restriction fragment length polymorphisms (RFLPs) and microsatellite markers, have been used to detect LOH through allelotypic comparisons of DNA from a cancer sample and a matched normal sample [98]. However, this approach is time consuming, and labor intensive, and requires a large amount of sample DNA, allowing only a modest number of markers to be screened. Most of the early LOH studies were focused on individual chromosomes, and only a few genome-wide LOH studies have been performed on NPC [34, 99–101]. The most frequent LOH were observed at chromosome 3p, 9p, and 14q, which is in agreement with the CGH based findings.

The mapping of the human genome has allowed for the identification of millions of SNP loci (http://www.ncbi.nlm.nih.gov/SNP), which makes them ideal markers for various genetic analyses, including LOH. Because of their abundance, even spacing, and stability across the genome, SNPs have significant advantages over RFLPs and microsatellite markers as a basis for high-resolution whole genome allelotyping with accurate copy number measurements. High-density oligonucleotide arrays have recently been generated to support large-scale high throughput SNP analysis [102]. It is now possible to genotype over 500,000 SNP markers using the Affymetrix Mapping 500K SNP oligonucleotide array. LOH patterns generated by SNP array analysis have a high degree of concordance with previous microsatellite analyses on the same cancer samples [103]. Additionally, shared regions of LOH from SNP arrays can cluster lung cancer samples into subtypes [104], and distinct patterns of LOH are found to associate with specific clinical features in primary breast, bladder, head and neck, and prostate tumors [93, 105–108]. While SNP array has not been utilized in NPC studies, it is expected that large scale SNP array-based LOH profiles will be generated on NPC in the near future. It is worth noting that a high-density SNP array is also a very powerful tool for identifying susceptibility gene(s) using either linkage or association study designs. One might envision that with a single high-throughput genomic platform, large-scale population-based study, searching for genetic susceptibility of NPC (inherited risk factors) can be performed concurrently with genomic profiling of NPC (somatic mutations).

**Cytogenetic analysis of NPC**

Cytogenetics has be widely used since the introduction of chromosome-banding techniques (keryotyping) in 1969 [109, 110]. One major drawback of these approaches is the requirement of in vitro culture and metaphase preparation of the cells of interest. Due to the poor tumor growth in vitro, only a limited number of karyotyping-based studies have been performed on primary NPC, which suggested genomic aberrations of 3q and 5q [111, 112]. Nevertheless, cytogenetic approaches will always have their place in the genomic profiling due to the ability to directly visualize chromosomal abnormalities. To obtain the cytogenetic information, cell lines and xenografts have been used frequently for the karyotyping studies on NPC, where many structural and numerical alterations found on 1p, 3p, 3q, 5q, 9p, 12, 11q, 13q, 14q, 16q, and X [113–118]. Among these alterations, deletion of 3p and gain of 3q are the most frequent events [119, 120]. More importantly, these cytogenetic techniques complement CGH and LOH by providing information on chromosomal structural rearrangements that are not resolved by DNA copy number analyses. For example, balanced translocations are one of the more common genomic abnormalities in cancer [121], but they cannot be detected by CGH or LOH. An experienced cytogeneticist, however, can readily detect many forms of chromosomal rearrangements of NPC using classical cytogenetic techniques, such as karyotyping [122].

The advances in the labeling techniques lead to the development of fluorescence in situ hybridization (FISH) method, which has proven to be an excellent choice for independent validation of other genomic methods. Fan et al. [123] reported FISH-based studies to validate the frequent amplification of c-myc and Int-2 that was initially discovered by CGH analysis. Recently, with the introduction of several new labeling techniques, such as spectral karyotyping (SKY), multicolor FISH (M-FISH), cross-species color banding (Rx-FISH), and multicolor chromosome banding, it is possible to carry out discovery studies using the cytogenetic methods. These techniques permit the simultaneous visualization of all chromosomes in different colors, and thus considerably improve the detection of translocations or deletions. For example, both SKY and M-FISH use a combinatorial labeling scheme with spectrally distinguishable fluorochromes. The chromosome-specific probe pools (chromosome painting probes) are generated from flow-sorted chromosomes and then amplified and fluorescently labeled by degenerate oligonucleotide-primed polymerase chain reaction. With the introduction of these techniques in 1996 [124–126], the comprehensive analysis of complex chromosomal rearrangements present in tumor karyotypes was greatly improved. A recent SKY analysis on NPC cell lines confirmed most of the abnormalities identified previously by CGH and LOH and illustrated additional breakpoints on a number of apparently balanced chromosomes, including 3p21, 3q26, 5q31, 6p21-p25, 7p14-p22 and 8q22 [127].

#### 2.2.2. Genome-wide expressional microarray analysis of NPC

The use of microarray and other global profiling technologies has led to a significant number of exciting new biological discoveries, and important correlations between gene-expression patterns and disease states. Never before could a small sample of RNA from two different conditions reveal so much information at the transcriptional level. Microarray-based expression profiling on tumor tissues have been used to identify molecular signatures that can promote the precise classification and prognostication of various types of cancers. Historically, only a few expression profiling analysis studies have been performed on NPC [128–130]. The limited amount of available clinical materials and heavy infiltration of non-cancer cells present major difficulties for these studies. With advances in preamplification technologies and microdissection tools, comprehensive expression profiling of NPC is possible. In the past couple of years, several genome-wide expression profiling studies have been devoted to identify candidate genes (e.g., genes involved in regulations of Ras activity, cell cycle, and WNT pathway) [131, 132], investigate the disease etiology (e.g., EBV infection, host responses, and hypoxia) [133–135], and evaluate the therapeutic effectiveness on NPC [136]. With the continuation of advances in genome-wide expressional microarray technology, comprehensive expression profiling of NPC is now starting to take the center stage. This should lead to substantial translational outcomes that will advance the management of this disease.

#### 2.2.3. Comprehensive genomic approaches

**Figure 2:**
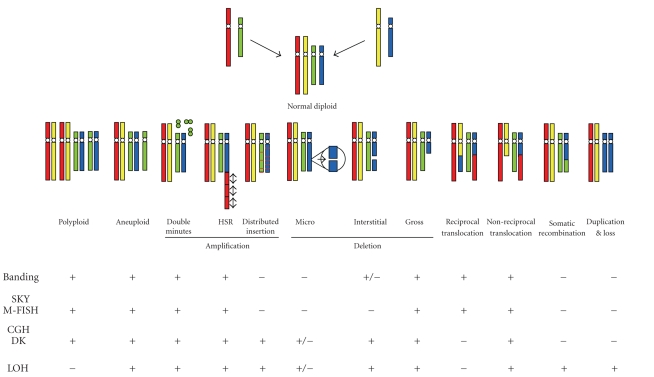
Identification of chromosomal abnormalities using various genomic and cytogenetic approaches. “+” and “−” denote effectiveness and ineffectiveness of the methods for the detection of a specific chromosomal abnormality. Banding: chromosome banding or karyotyping analysis; SKY: spectral karyotyping analysis; M-FISH: multicolor fluorescence in situ hybridization; CGH: comparative genomic hybridization; DK: digital karyotyping analysis; LOH: loss of heterozygosity. Adapted from [137] with kind permission of Future Drugs Ltd.

A major challenge confronting the identification of the molecular genetic factors that contribute to the NPC tumorigenesis is the diversity of the genetic alterations. Among these are germline variations (such as the susceptibility genes described in previous section) that lead to hereditary cancer predispositions, the acquisition of transforming DNA or RNA sequences from cancer viruses (e.g., EBV for NPC), somatic mutations in the cancer genome (e.g., copy number change, translocation, LOH), and epigenetic mechanisms (such as DNA methylation or histone modification) that promote oncogenesis by modifying cancer-related genes. Somatic genomic alterations such as point mutations, genomic amplifications or deletions, loss of allelic heterozygosity, and chromosomal translocations are believed to play a central role in the development of most solid tumors, including NPC. All of these mechanisms result in dysregulated expression of oncogenes and tumor suppressor genes, but none of the existing genomic techniques can capture all of these genetic changes in a single analysis (see Figure 2). This represents a major obstacle to the comprehensive analysis of tumor genomes and their relationship to clinical phenotypes or disease progression.

A more practical approach to overcome this problem is to combine a selective set of molecular genetic technologies such as CGH, LOH, and various molecular cytogenetic analyses for comprehensive screening of genomic alterations with high resolution. Each of these techniques has their own unique advantages, but they also have their own limitations which have motivated efforts to combine these approaches as shown in Figure 2. In this instance, the SNP array-based LOH and CGH analyses provide a high-resolution mapping of copy number abnormalities, but offer little information on chromosomal structure/spatial changes (e.g., translocations, the most common class of somatic mutation registered in the cancer gene census [121]). On the other hand, modern cytogenetic techniques provide a clear picture of the gross chromosomal structure/spatial alterations, but have limited resolution. Therefore, strategically combining a complementary set of genetic tests is a logical approach for characterizing a complex cancer genome. This has been successfully attempted to investigate the immortalization of nasopharyngeal epithelial cells [138], where karyotyping, spectral karyotyping (SKY) and array CGH were utilized concurrently to reveal a gain of 17q21–q25 fragment on 11p15 chromosome, with the specific derivative chromosome 11: der(11)t(11;17)(p15.1;q21.1).

This multimodal approach can be extended to combine DNA structural analyses with additional genome functional activity at the RNA and/or protein levels. Recent technical advances in microarray-based gene expression analysis have offered substantial improvement in diagnosis, treatment, and prognosis of cancer patients. This continuous progress in microarray-based expression analysis and the large public depositories of microarray data have motivated new efforts to extract additional biological information from these data in addition to the static RNA transcript levels. One such attempt involves inferring the chromosomal structural changes from spatially-linked changes in microarray expression data [139–142]. Several array CGH studies have shown a genome-wide correlation of gene expression with copy number alterations and have proved useful in individual amplicon refinement [143, 144]. For example, through tissue microarray FISH and RT-PCR, a minimally amplified region around ERBB2 was identified in a large number of breast tumors. In addition, gene amplification was found to be correlated with increased gene expression in a subset of those samples [145]. Recently, several groups have observed that chromosomal alterations can lead to regional gene expression biases in human tumors and tumor-derived cell lines [139–141, 146, 147]. A recent study also demonstrated the correlation between SNP array-based LOH profiles and expression profiles [105]. These studies suggest that a fraction of gene expression values (15–25%) are regulated in concordance with chromosomal DNA content [139–141, 146, 147]. Several statistical methods have been developed and have shown promising results for detecting DNA copy number abnormalities based on differential gene expression [139–142]. With the recent growth in transcriptomic profiling studies of NPC, these techniques for “reverse inference” of DNA alterations from RNA expression data will become a valuable approach for genomic profiling that can provide cross-validation of functional genomic alterations at multiple biological levels when combined with DNA-based approaches such as CGH and LOH. These attempts for strategic integration of genomic information at multiple levels provide an exciting paradigm to introduce the system biology (or more specifically system genomics) concept into NPC research. Further strategies for implementing a comprehensive database that contains additional levels of genomic information such as alternative splicing and methylation status have also been suggested.

## 3. FUTURE DIRECTIONS

The high susceptibility of individuals in Southern Asia to NPC is still puzzling. The recent advances in the single nucleotide polymorphism and haplotype analyses, genome-wide screening, and association studies may help to decipher the inheritable genetic components for this enigmatic cancer. The cellular genes involved in DNA damage and its association with EBV entry or latency should be focused upon and further explored. Recently deployed technologies, such as high-density SNP array, will play a critical role in the search of these susceptibility genes. This same platform has also been successfully adapted to perform LOH and CGH profiling of the cancer genome, which place it in a unique position in the area of NPC research.

Previous molecular studies on NPC have focused on DNA and chromosomal levels, but few on transcriptomic and proteomic profiles. Small biopsy material and heavy infiltration of non-cancer cells present major difficulties for transcriptomic and proteomic studies. With advances in microdissection and preamplification technology, comprehensive expression profiling of NPC is now starting to take center stage. This should lead to substantial translational outcomes that will advance the management of this disease.

While substantial amount of information on the genomic alteration of NPC have been accumulated, the recent advances in genomic technologies (e.g., high-density SNP array) and the vast resources created by Human Genome Project will lead to more comprehensive results. Strategic integration of the data streams from multiple experimental applications (e.g., CGH, LOH, and expression microarray) at different biological levels (e.g., DNA, RNA and protein levels) will greatly enhance our ability to capture the precise portrait of the NPC genome.

## Figures and Tables

**Table 1:** The most frequent genomic abnormalities of NPC.

	Frequent abnormalities
CGH	Gain: 1q, 3q, 8q, 12p, 12q and loss: 3p, 9p, 11q, 14q, 16q
LOH	3p, 9p, and 14q,
Karyotyping	Gain: 3q and loss: 3q
